# The Relationship Between Mother Narrative Style and Child Memory

**DOI:** 10.4021/jocmr613w

**Published:** 2011-07-26

**Authors:** Sinan Mahir Kayιran, Sena Cure

**Affiliations:** a Department of Pediatrics, American Hospital, Istanbul, Turkey; b Psychology Department, Koc University, Istanbul, Turkey

## Abstract

**Background:**

The question of whether children and infants have memory capabilities similar to adults has long been of interest. Until recently, it was thought that compared to adults, infants have very limited memory processing abilities. Knowledge about factors affecting a child's memory abilities can help families (specifically mothers) behave in a manner that best benefits their children in language and memory skills. The present study examines one factor that may underlie a child's memory capabilities; namely the mother's narrative style.

**Methods:**

Convenience sampling was used to select participants. Forty healthy children (mean age of 31.55 months, range 25-37 months) and their mothers were entered into the study. All participants were native Turkish speakers, from similar socioeconomic status backgrounds. Memory was assessed by a modified version of the Magic Shrinking Machine. Narrative style was assessed by the mother "reading" a Frog Story; a picture book with no words in it. Children were then grouped according to their mother's level of narrative style. Children's language skills were measured via the Turkish form of the CDI (Communicative Development Inventory) which was translated to Turkish as TIGE.

**Results:**

To explore the relationships between mothers' narrative styles and children's memory and language skills and between children's language skills and memory capabilities, linear regressions were run. There were no significant correlations among any comparisons (*P* > 0.05).

**Conclusions:**

Children's language skills do not improve according to their mothers' narrative styles, and children do not show better memory abilities when mothers use more words and longer sentences. In order to have a better understanding of these relationships, future research that includes several more variables is needed.

**Keywords:**

Child; Mother; Memory; Narrative style

## Introduction

The question of whether children and infants have memory capabilities similar to adults has long been of interest. Until recently, it was thought that compared to adults, infants have very limited memory processing abilities. However, current research shows that children as young as two years old remember events from their daily lives [1, 2]. The factors involved in infant memory processing ability and the variables leading to differences between children in memory processing are not yet understood. Knowledge about factors affecting a child's memory abilities can help families (specifically mothers) behave in a manner that best benefits their children in language and memory skills. The present study examines one factor that may underlie a child's memory capabilities; namely the mother's narrative style.

Narrative style includes how a mother uses language to talk to their child, and when she communicates with her child. Long and complex sentences may have a very different affect than short and simple sentences on a child's memory or more specifically, how well the child can remember an event in which he/she participated. However, the child's language skills may be a confounding variable in this relationship.

The main question investigated in the present study is whether a mother's narrative style affects her child's memory. It is hypothesized that, children of mothers who use many different words and longer sentences will have better memory capabilities than children whose mothers use less words and shorter sentences. It is also predicted that a mother's narrative style will affect her child's language skills, and subsequently, the child's language skills will affect memories.

## Material and Methods

Convenience sampling was used to select participants. Forty healthy children (mean age of 31.55 months, range 25-37 months) and their mothers were entered into the study. All children were recruited from the well-child clinic in the pediatrics department of the American Hospital in Istanbul which generally services patients from high socio-economic status families. Data from one child who did not complete the study were dropped and thus the final sample size was 39 (21 girls and 18 boys). A written informed consent was received from all mothers. All participants were native Turkish speakers, from similar socioeconomic status backgrounds. Memory was assessed by a modified version of the Magic Shrinking Machine [1]. Narrative style was assessed by the mother "reading" a Frog Story; a picture book with no words in it. Children were then grouped according to their mother's level of narrative style. Children's language skills were measured via the Turkish form of the CDI (Communicative Development Inventory) which was translated to Turkish as TIGE. TIGE-1 was designed to assess the language development of children between 8 and 16 months and TIGE-2 for 16-36 months. The scale is effective in screening language delay and language disorders. It has two different forms for different age levels [3]. The TIGE assessment was completed by the mothers [4].

Participants were tested twice in their homes. During the first visit, the Magic Cutting Task was completed. In this task, a green piece of cardboard was shown to the children. The cardboard was then cut in a specific way with bunny-shaped scissors as to make a hole in the cardboard. Every attempt was made to get the child to participant in this event. While the researcher was cutting the cardboard, a picture of a magician was given to the child for him/her to color. During this initial visit the mothers were also surveyed and the TIGE II form was completed.

The second visit took place approximately 1 month after the first. Memory for the Magic Cutting Task was assessed mainly through verbal recall (e.g. "Do you remember me?", "Ok, we played a game, what else did we do?"). Photograph identification (Showing 6 pictures - "While I was cutting, which one of these pictures did I give to you, can you show me?") and behavioral reenactment ("Where did we play? Take me there.") were also used to assess memory [1].

The primary goal of the study was to investigate the effect of mother narrative style on children's memory. In addition, we also explored the effect of mother narrative style on children's language skills, and the affect of language skills on children's memory. All the statistical relationships were analyzed first by correlation, then by regression analysis in SPSS (Statistical Package for Social Scientists) Version 17 for Windows. One-tailed bivariate correlations were run between narrative style and memory, narrative style and language skills, and language skills and memory. *P* < 0.05 was accepted as significant.

## Results

There were 14 mothers who participated in the Frog Story task (assessing narrative style). Forty children participated in the memory task (Magic Cutting Task) during the second home visit, and thirty-nine mothers completed the TIGE II forms about their child's language skills. Descriptive statistics of narrative styles, memory and language skills are shown in Table 1.

**Table 1:** Descriptive Statistics of Mothers' Narrative Style, Children’s Memory, and Total Children's Language Skill Score

	*n*	Mean (*SD*)	*CI*
Mothers' narrative style	14	13.14 (3.70)	8 - 23
Children's memory	40	20.08 (12.54)	1 - 52
Children's language skills	39	638.95 (140.12)	140 - 819

SD: standard deviation; CI: confidence interval.

To explore the relationships between mothers' narrative styles and childrens' memory and language skills and between children's language skills and memory capabilities, linear regressions were run. Correlation results are shown in Table 2. There were no significant correlations among any comparisons (*P* > 0.05).

**Table 2:** Correlation Between Mothers' Narrative Style and Children's Memory, Mothers' Narrative Style and Children's Language Skills, and Children's Language Skills and Memory

		Mothers' narrative styles (*n* = 14)	Children's memory (*n* = 40)	Children's language skills (*n* = 39)
Mothers' narrative styles	Pearson Correlation	1	-0.189	0.004
	*P* (1-tailed)		0.259	0.495
Children's memory	Pearson Correlation	-0.189	1	-0.032
	*P* (1-tailed)	0.259		0.424
Children's language skills	Pearson Correlation	0.004	-0.032	1
	*P* (1-tailed)	0.495	0.424	

The proposed hypotheses were proven false. Children's language skills do not improve according to their mothers' narrative styles, and children do not show better memory abilities when mothers use more words and longer sentences.

## Discussion

The aim of the present study was to examine the relationships between mothers' narrative styles and children's memory abilities and language skills. The results found no relationship between any variable studied. There was no correlation between a mother's narrative style and child's memory or language skills. In addition, a child's language skills were not significantly correlated with memory ability.

There are many possible explanations for the negative findings. Children between the ages of two and two and a half do not attend to verbal information as much as adults. Children rely instead on nonverbal information. Thus, it may have been necessary for the children in the present study to translate nonverbal information into verbal information when reporting about a past event; a task requiring a well-developed language capability. Simcock and Hayne [1] found that children are not skilled in the translation of nonverbal information into verbal information. They discovered however, that interaction with a mother providing rich narrations greatly assisted children in this task. A mother's verbalizations provide children with a greater vocabulary, increasing their verbal memory abilities. The importance of word familiarity was demonstrated by [5-7]. The mothers in the present study did not aid their children during the Magic Cutting Task. It is possible that a correlation may have been found if mothers were allowed to narrate to their children during this task.

There are a number of limitations in the interpretation of results from the present study. Not all children who participated in this study were excited about answering the memory questions, so the data that is used in this research may not be a good representative of 2-year-old's memory. In addition, the sampling method was not random but of convenience and the sample size was small and homogenous in that family backgrounds were socioeconomically similar. The one month interval between sessions may also have been a confounding variable. The experiences during the interval most likely varied between children. Some children may have been exposed to experiences that may influence subsequent performance on the memory task. For example, some children may have encountered a similar task in the one month interval and may therefore perform better. On the other hand, it is possible that a child who had been exposed to a task similar to the Magic Cutting Task may answer questions at the second session that relate to the interim task experience.

The negative findings from this study are important in the area of child memory and language development. Previous studies have shown significant effects between child memory and child language development [8]. In addition, research has shown that, conversation styles of mothers impact immediate and long-term memory of children [9-11]. More recently, Reese and Newcombe [12] trained a group of mothers to others use more words while interacting with their children. Children of trained mothers demonstrated richer and more accurate memories than children of untrained mothers. The discrepancy between our results and results from prior studies investigating similar variables is intriguing and may ultimately be due to some limitations of the present study. Repeating the present study with an increased sample size may raise the significance level of our results. The age of the children in the present study may also be a factor in the negative results. Two years old may be too young to assess the relationship between memory and mother narrative style. This study should be repeated with older children. In order to have a better understanding of these relationships, future research that includes several more variables is needed.
